# Polymorphisms of rs1347093 and rs1397529 are associated with lung cancer risk in northeast Chinese population

**DOI:** 10.18632/oncotarget.22030

**Published:** 2017-10-24

**Authors:** Xiaoying Li, XueLian Li, Yangwu Ren, Zhihua Yin, Xiaowei Quan, Xiaoxia Xue, Baosen Zhou

**Affiliations:** ^1^ Department of Epidemiology, School of Public Health, China Medical University, Shenyang, China; ^2^ Key Laboratory of Cancer Etiology and Prevention (China Medical University), Liaoning Provincial Department of Education, Liaoning, China; ^3^ The Third Center of Laboratory Technology and Experimental Medicine, China Medical University, Shenyang, China

**Keywords:** single nucleotide polymorphisms, MIR217HG, Gab1, lung cancer, susceptibility

## Abstract

Lung cancer is one of the malignant tumors with the highest morbidity and mortality all over the world. Here we researched the association between two SNPs (rs1347093 in MIR217HG and rs1397529 in Gab1) and the risk of lung cancer in northeast Chinese population, including 825 cases and 766 controls. We carried out χ^2^ test, unconditional logistic regression analysis and crossover analysis to estimate the relationship between SNPs and lung cancer risk and the interaction between SNPs and smoking on susceptibility to lung cancer. The results indicated that rs1347093, rs1397529 polymorphisms were associated with lung cancer risk, especially with adenocarcinoma risk. Dominant genetic model of the rs1347093 was associated with reduced risk of lung cancer compared to CC genotype (AC+AA vs. CC: adjusted OR = 0.599, 95%CI = 0.418-0.858, *P=*0.005). For rs1347093, the similar result was found. Dominant genetic model of the rs1397529 was associated with reduced risk of lung cancer compared to AA genotype (AC+CC vs. AA: adjusted OR = 0.664, 95%CI = 0.491-0.897, *P=*0.008). There is no significant interaction between rs1347093, rs1397529 polymorphism and smoking on susceptibility to lung cancer. Our study might demonstrate that rs1347093 in MIR217HG and rs1397529 in Gab1 could be meaningful as the novel biomarker for lung cancer risk.

## INTRODUCTION

According to the latest cancer statistics, lung cancer is one of the malignant tumors with the highest morbidity and mortality all over the world [1–3]. Approximate 14.1 million new cancer cases occurred in 2012, including about 1.8 million lung cancer cases, which constituted about 12.8% of all new cancer cases [3]. As we all know, the leading risk factor for lung cancer is smoking [4]. However, more and more studies indicate that genetic risk factors may also play an important part in the occurrence and development of lung cancer [5–7]. Meanwhile, the interaction between genes and smoking may exist. Therefore, this study intended to explore the association between single nucleotide polymorphisms (SNPs) and the susceptibility of lung cancer, and the interaction between SNPs and smoking on lung cancer risk.

MIR217HG (MIR217 host gene) is located in 2p16. It is the host gene of MIR217. Some previous studies demonstrate that MIR217 is closely related to the occurrence and development of tumor. For example, N. A. Schultz et al [8] proved the diagnostic MIR217 expression profile associated with pancreatic cancer, which was described by Szafranska et al [9]. However, up to now, there are no reports about the function of MIR217HG and the association between MIR217HG and any diseases.

The Gab-family adapter proteins are also known as the Grb2-associated binder family adapter proteins, which are scaffolding adapter molecules [10]. Gab proteins mainly consist of Gab1, Gab2 and Gab3 [11]. The weights of Gab1 molecules are 100-120KD [12, 13]. Gab1 is located in 4q31 and it most mainly exists in brain, kidney, lung, heart, testis, and ovary [13, 14]. According to previous studies [13, 15], Gab1 worked in EGF receptor / ErbB-mediated cell transformation, which indicated that Gab1 played an important role in oncogenic signaling pathways. Fan Y et al [16] found that Gab1 had the overexpression in chondrosarcoma tissues, which made Gab1 be considered as a novel biomarker of diagnosis and prognosis of chondrosarcoma. The study of Bai R et al [17] indicated that Gab1 played a role in the function exertion of miR-409-3p, which was considered as a metastatic suppressor. However, to the best of my knowledge, no study about the association between Gab1 and lung cancer was carried out.

Given that the relationship between MIR217HG, Gab1 and lung cancer was undefined, the present study evaluated the relationship between MIR217HG-rs1347093, Gab1-rs1397529 polymorphisms and lung cancer susceptibility and investigated the interaction between MIR217HG-rs1347093, Gab1-rs1397529 polymorphisms and smoking status on the risk of lung cancer, which could provide a new strategy of lung cancer screening in Chinese population.

## RESULTS

### Baseline characteristics

The demographic characteristics of 825 cases and 766 controls were shown in Table 1. In case group, there were 483 cases of adenocarcinoma, 207 cases of squamous cell carcinoma, 95 cases of small cell lung cancer and 40 cases of other histological type. There was no significant difference in the distribution of age (*P* = 0.197) between lung cancer patients and controls with the mean age for cases and controls were 58.567 ± 11.054 and 57.158 ± 13.341 respectively. However, there was a significant difference in the distributions of gender and smoking (both were *P<*0.001). Therefore, the further study was adjusted by gender, age and smoking.

**Table 1:** Demographics of the study subjects

Characteristics	Cases n(%)	Controls n(%)	*P*
Gender			<0.001
Female	619(75.0)	658(85.9)	
Male	206(25.0)	108(14.1)	
Age (years)	58.567±11.054	57.158±13.341	0.197
Stage^*^			
I	52(7.8)		
II	132(19.9)		
III	331(49.9)		
IV	149(22.4)		
Histological type			
Adenocarcinoma	483(58.5)		
Squamous cell carcinoma	207(25.1)		
Small cell lung cancer	95(11.5)		
other	40(4.9)		
Family history of cancer^*^			0.912
yes	68(12.6)	82(12.8)	
no	471(87.4)	557(87.2)	
Smoking			<0.001
yes	183(22.2)	54(7.0)	
no	642(77.8)	712(93.0)	

^*^There are missing values.

### The distributions of genotypes, alleles and associations with lung cancer

Distributions of rs1347093, rs1397529 genotypes and alleles among subjects and their associations with the risk of lung cancer were summarized in Table 2. The observed genotype frequencies of rs1347093, rs1397529 among controls was in agreement with Hardy-Weinberg equilibrium (χ^2^ = 0.984, *P =* 0.321 for rs1347093; χ^2^ = 0.154, *P =* 0.695 for rs1397529). For the distribution of rs1347093, A allele had the relationship with a significantly reduced risk of lung cancer (adjusted OR = 0.619, 95%CI = 0.436-0.877, *P=*0.007), compared with C allele. Meanwhile, AC genotype of the rs1347093 was linked to reduced risk of lung cancer compared to CC genotype (AC vs. CC: adjusted OR = 0.594, 95%CI = 0.413-0.853, *P=*0.005). Besides, we observed that a significant association between the rs1347093 polymorphism and reduced risk of lung cancer existed in a dominant genetic model, assuming that the variant genotypes (AC+AA) was the dominant genetic model (AC+AA vs. CC: adjusted OR = 0.599, 95%CI = 0.418-0.858, *P=*0.005). For the distribution of rs1397529, C allele had the relationship with a significantly reduced risk of lung cancer (adjusted OR = 0.697, 95%CI = 0.526-0.926, *P=*0.013), compared with A allele. Meanwhile, a significant association between the rs1397529 polymorphism and reduced risk of lung cancer in one genetic model could be observed (AC vs. AA: adjusted OR = 0.649, 95%CI = 0.476-0.884, *P=*0.006). Besides, we observed that a significant association between the rs1397529 polymorphism and reduced risk of lung cancer existed in a dominant genetic model, assuming that the variant genotypes (AC+CC) was the dominant genetic model (AC+CC vs. AA: adjusted OR = 0.664, 95%CI = 0.491-0.897, *P=*0.008).

**Table 2:** Distributions of rs1347093, rs1397529 genotypes and alleles among lung cancer cases and controls and their associations with the risk of lung cancer

Genotype	Cases n(%)	Controls n(%)	*P*	OR [5]	*P* adj	OR[95%CI] adj
rs1347093						
CC	768(93.1)	680(88.8)		1.00(ref)		1.00(ref)
AC	56(6.8)	85(11.1)	0.003	0.583(0.410,0.830)	0.005	0.594(0.413,0.853)
AA	1(0.1)	1(0.1)	0.931	0.885(0.055,14.183)	0.978	1.040(0.065,16.667)
AC/AA	57(6.9)	86(11.2)	0.003	0.587(0.413,0.833)	0.005	0.599(0.418,0.858)
C	1592(96.5)	1445(94.3)		1.00(ref)		1.00(ref)
A	58(3.5)	87(5.7)	0.004	0.605(0.431,0.850)	0.007	0.619(0.436,0.877)
rs1397529						
AA	736(89.2)	646(84.3)		1.00(ref)		1.00(ref)
AC	83(10.1)	114(14.9)	0.004	0.639(0.473,0.864)	0.006	0.649(0.476,0.884)
CC	6(0.7)	6(0.8)	0.822	0.878(0.282,2.735)	0.944	0.959(0.303,3.039)
AC/CC	89(10.8)	120(15.7)	0.004	0.651(0.485,0.873)	0.008	0.664(0.491,0.897)
A	1555(94.2)	1406(91.8)		1.00(ref)		1.00(ref)
C	95(5.8)	126(8.2)	0.006	0.682(0.517,0.898)	0.013	0.697(0.526,0.926)

*P* adj and OR[95%CI] adj were adjusted for age, gender and smoking.

### Stratification analyses

In order to further study the relationship between rs1347093, rs1397529 polymorphism and lung cancer risk, we conducted several stratification analyses in the dominant genetic model. As shown in Table 3, AA/AC genotype of rs1347093 had the relationship with a significantly decreased risk of lung cancer in adenocarcinoma subgroup (adjusted OR = 0.598, 95%CI = 0.393-0.912, *P=*0.017) and small cell lung cancer subgroup (adjusted OR = 0.296, 95%CI = 0.106-0.826, *P=*0.020), compared with CC genotype. There was no significant association between the dominant genetic model of rs1347093 and the susceptibility of lung cancer in squamous cell carcinoma subgroup. For rs1397529, CC/AC genotype was linked with a significantly decreased risk of lung cancer in adenocarcinoma subgroup (adjusted OR = 0.689, 95%CI = 0.488-0.974, *P=*0.035), compared with AA genotype. We failed to observe a significant association between the dominant genetic model of rs1397529 and the susceptibility of lung cancer in squamous cell carcinoma subgroup and small cell lung cancer subgroup.

**Table 3:** The effect of the SNPs rs1347093 and rs1397529 in the region on lung cancer risk in pathological subgroup

Genotype	Cases n(%)	Controls n(%)	*P* value	OR(95%CI)	*P* adj	OR(95%CI) adj
rs1347093						
Adenocarcinomas						
CC	450(93.2)	680(88.8)		1.00(ref)		1.00(ref)
AA/AC	33(6.8)	86(11.2)	0.011	0.580(0.382,0.881)	0.017	0.598(0.393,0.912)
Squamous cell carcinoma						
CC	188(90.8)	680(88.8)		1.00(ref)		1.00(ref)
AA/AC	19(9.2)	86(11.2)	0.400	0.799(0.474,1.347)	0.522	0.826(0.460,1.484)
Small cell lung cancer						
CC	91(95.8)	680(88.8)		1.00(ref)		1.00(ref)
AA/AC	4(4.2)	86(11.2)	0.015	0.280(0.101,0.778)	0.020	0.296(0.106,0.826)
rs1397529						
Adenocarcinomas						
AA	429(88.8)	646(84.3)		1.00(ref)		1.00(ref)
AC/CC	54(11.2)	120(15.7)	0.026	0.678(0.481,0.955)	0.035	0.689(0.488,0.974)
Squamous cell carcinoma						
AA	186(89.9)	646(84.3)		1.00(ref)		1.00(ref)
AC/CC	21(10.1)	120(15.7)	0.047	0.608(0.372,0.994)	0.171	0.686(0.401,1.176)
Small cell lung cancer						
AA	87(91.6)	646(84.3)		1.00(ref)		1.00(ref)
AC/CC	8(8.4)	120(15.7)	0.066	0.495(0.234,1.048)	0.084	0.515(0.242,1.093)

*P* adj and OR[95%CI] adj were adjusted for age, gender and smoking.

Table 4 summarized the associations between rs1347093, rs1397529 polymorphism and lung cancer risk, stratified by clinical stages. We could obtain a significant association between AA/AC genotype of rs1347093 and reduced risk of lung cancer in comparison with CC genotype in stage III+ IV subgroup (adjusted OR = 0.568, 95%CI = 0.368-0.876, *P=*0.011). Meanwhile, we also observed that CC/AC genotype of rs1397529 was associated with reduced risk of lung cancer in comparison with AA genotype in stage III+ IV subgroup (adjusted OR = 0.647, 95%CI = 0.452-0.925, *P=*0.017).

**Table 4:** Associations between rs134709, rs1397529 polymorphisms and lung cancer susceptibility, stratified by stages

Genotype	Cases n(%)	Controls n(%)	*P*	OR(95%CI)	*P adj*	OR(95%CI) adj
rs1347093						
stage I+II						
CC	169(91.8)	680(88.8)		1(ref)		1(ref)
AA/AC	15(8.2)	86(11.2)	0.226	0.702(0.395,1.246)	0.232	0.700(0.390,1.256)
stage III+ IV						
CC	449(93.5)	680(88.8)		1(ref)		1(ref)
AA/AC	31(6.5)	86(11.2)	0.006	0.546(0.356,0.837)	0.011	0.568(0.368,0.876)
rs1397529						
stage I+II						
AA	165(89.7)	646(84.3)		1(ref)		1(ref)
AC/CC	19(10.3)	120(15.7)	0.068	0.620(0.371,1.036)	0.111	0.655(0.390,1.102)
stage III+ IV						
AA	431(89.8)	646(84.3)		1(ref)		1(ref)
AC/CC	49(10.2)	120(15.7)	0.007	0.612(0.430,0.872)	0.017	0.647(0.452,0.925)

*P* adj and OR[95%CI] adj were adjusted for age, gender and smoking.

Table 5 showed the associations between rs1347093, rs1397529 polymorphism and lung cancer risk, stratified by smoking. We observed a significant association between AA/AC genotype of rs1347093 and reduced risk of lung cancer in both smoking subgroup and non-smoking subgroup, compared with CC genotype (smoking subgroup: adjusted OR = 0.664, 95%CI = 0.491-0.897, *P=*0.008; non-smoking subgroup: adjusted OR = 0.559, 95%CI = 0.380-0.823, *P=*0.003). We also observed that CC/AC genotype of rs1397529 was linked to reduced risk of lung cancer in non-smoking subgroup, compared with AA genotype (adjusted OR = 0.595, 95%CI = 0.430-0.822, *P=*0.002).

**Table 5:** Associations between rs1347093, rs1397529 polymorphisms and lung cancer susceptibility, stratified by smoking status

Genotypes	Cases n(%)	Controls n(%)	*P*	OR[95%CI]	*P* adj	OR[95%CI] adj
rs1347093						
Non-exposed						
CC	599(93.3)	630(88.5)		1(ref)		1(ref)
AC/AA	43(6.7)	82(11.5)	0.002	0.552(0.375,0.811)	0.003	0.559(0.380,0.823)
Exposed						
CC	169(92.3)	50(92.6)		1(ref)		1(ref)
AC/AA	14(7.7)	4(7.4)	0.004	0.651(0.485,0.873)	0.008	0.664(0.491,0.897)
rs1397529						
Non-exposed						
AA	576(89.7)	596(83.7)		1(ref)		1(ref)
AC/CC	66(10.3)	116(16.3)	0.001	0.589(0.426,0.813)	0.002	0.595(0.430,0.822)
Exposed						
AA	160(87.4)	50(92.6)		1(ref)		1(ref)
AC/CC	23(12.6)	4(7.4)	0.300	1.797(0.593,5.443)	0.301	1.800(0.591,5.478)

*P* adj and OR[95%CI] adj were adjusted for age and gender.

Table 6 listed the associations between rs1347093, rs1397529 polymorphism and lung cancer risk, stratified by gender. We observed a significant association between AA/AC genotype of rs1347093 and reduced risk of lung cancer in comparison with CC genotype in the female subgroup (adjusted OR = 0.582, 95%CI = 0.391-0.866, *P=*0.008). We also found that CC/AC genotype of rs1397529 was associated with reduced risk of lung cancer in comparison with AA genotype in the female subgroup (adjusted OR = 0.605, 95%CI = 0.432-0.847, *P=*0.003).

**Table 6:** Associations between rs1347093, rs1397529 polymorphisms and lung cancer susceptibility, stratified by gender

Genotypes	Cases n(%)	Controls n(%)	*P*	OR[95%CI]	*P* adj	OR[95%CI] adj
rs1347093						
Female						
CC	576(93.1)	584(88.8)		1(ref)		1(ref)
AC/AA	43(6.9)	74(11.2)	0.008	0.589(0.398,0.873)	0.008	0.582(0.391,0.866)
Male						
CC	192(93.2)	96(88.9)		1(ref)		1(ref)
AC/AA	14(6.8)	12(11.1)	0.192	0.583(0.260,1.310)	0.360	0.665(0.278,1.592)
rs1397529						
Female						
AA	556(89.8)	555(84.3)		1(ref)		1(ref)
AC/CC	63(10.2)	103(15.7)	0.004	0.611(0.437,0.853)	0.003	0.605(0.432,0.847)
Male						
AA	180(87.4)	91(84.3)		1(ref)		1(ref)
AC/CC	26(12.6)	17(15.7)	0.446	0.773(0.399,1.498)	0.993	0.997(0.486,2.045)

*P* adj and OR[95%CI] adj were adjusted for age and smoking.

### Interaction between rs1347093, rs1397529 and smoking

The results of crossover analysis were listed in Tables 7 and 8. We could find that there was no significant interaction between rs1347093, rs1397529 and smoking on lung cancer risk. The logistic regression model also demonstrated that interaction didn’t exist between rs1347093, rs1397529 and smoking on risk of lung cancer (Table 9).

**Table 7:** Interaction between rs1347093, rs1397529 polymorphisms and smoking on lung cancer susceptibility

SNP	Smoking	Cases	Controls	*P*	OR(95%CI)	*P* adj	OR(95%CI) adj
rs1347093							
AA/AC	Non-exposure	43	82		1(ref)		1(ref)
CC	Non-exposure	599	630	0.002	1.813(1.233,2.666)	0.003	1.785(1.212,2.628)
AA/AC	Exposure	14	4	0.001	6.674(2.070,21.524)	<0.001	9.202(2.704,31.318)
CC	Exposure	169	50	<0.001	6.446(3.966,10.474)	<0.001	9.007(4.877,16.637)
rs1397529							
CC/AC	Non-exposure	66	116		1(ref)		1(ref)
AA	Non-exposure	576	596	0.001	1.699(1.230,2.347)	0.002	1.679(1.215,2.322)
CC/AC	Exposure	23	4	<0.001	10.106(3.351,30.480)	<0.001	13.885(4.344,44.385)
AA	Exposure	160	50	<0.001	5.624(3.628,8.718)	<0.001	7.723(4.364,13.668)

*P* adj and OR[95%CI] adj were adjusted for age and gender.

**Table 8:** Crossover analysis of interaction between rs1347093, rs1397529 polymorphisms and smoking on lung cancer susceptibility

Measure	Estimate	Lower	Upper
rs1347093			
RERI	-0.975	-11.307	9.356
AP	-0.108	-1.255	1.038
S	0.891	0.282	2.814
rs1397529			
RERI	-6.845	-15.522	1.832
AP	-0.886	-2.899	1.126
S	0.495	0.146	1.686

**Table 9:** Logistic model of interaction between rs1347093, rs1397529 polymorphisms and smoking on lung cancer susceptibility

Variables	Cases	Controls	*P adj*	OR(95%CI) adj
rs1347093				
SNP				
AC/AA	57	86		1(ref)
CC	768	680	0.003	1.785(1.212,2.628)
smoking				
no	471	557		1(ref)
yes	68	82	<0.001	9.202(2.704,31.318)
interaction			0.334	0.548(0.162,1.857)
rs1397529				
SNP				
AC/CC	89	120		1(ref)
AA	736	646	0.002	1.679(1.215,2.322)
smoking				
no	471	557		1(ref)
yes	68	82	<0.001	13.885(4.344,44.385)
interaction			0.061	0.331(0.104,1.052)

*P* adj and OR[95%CI] adj were adjusted for age and gender.

## DISCUSSION

In recent years, more and more studies about the association between SNPs and lung cancer risk were conducted. The studies of Chen D [18], Xie K [19], Yin Z [20] et al all came to a significant conclusion. SNPs play an important role in the occurrence, development and prognosis of lung cancer [21]. In the present study, we evaluated the association between rs1347093, rs1397529 polymorphisms and lung cancer susceptibility by the statistical method in 825 cases and 766 controls. The results of the study indicated that rs1347093, rs1397529 polymorphisms were associated with lung cancer risk, especially with adenocarcinoma risk. For rs1347093 in MIR217HG, the individuals who carry the dominant genetic model (AC+AA) have less risk of lung cancer than those who carry CC genotype. For rs1397529 in Gab1, the individuals who carry the dominant genetic model (AC+CC) have less risk of lung cancer than those who carry AA genotype. In stratification analyses by histological type, we found that the individuals who carry the dominant genetic model of rs1347093, rs1397529 have less risk of adenocarcinoma, compared with those individuals who don’t carry the dominant genetic model of rs1347093, rs1397529. For rs1347093, the similar result was found in small cell lung cancer subgroup. The individuals who carry the dominant genetic model (AC+CC) have less risk of small cell lung cancer than those who carry AA genotype. The stratification analysis showed that the association of rs1347093, rs1397529 to risk of lung cancer was mainly derived from female. This may be on account of differences of men and women in the physiological and genetic aspects. We will research this issue further in the later study. We also researched interaction between rs1347093, rs1397529 and smoking on lung cancer risk. However, the results indicated that the interaction didn’t exist. One of the reasons may be that the number of subjects with the exposure of tobacco is not large enough to get a statistically significant difference.

rs1347093 is located in the intron region of MIR217HG, and rs1397529 is located in the UTR-3 region of Gab1. Although they can’t code proteins, they might have regulating effects, which might have an effect on gene function. Thus rs1347093, rs1397529 polymorphisms might influence the function of genes and might have the relationship with diseases. The function of rs1347093 and rs1397529 will be studied in our future study. Gab1 is a docking protein, which can transduce signals from all various tyrosine kinases [22]. Expression of Gab1 is associated with many cancer, such as ovarian cancer [23], breast cancer [22], chondrosarcoma [16], colorectal cancer [17], urothelial cell carcinoma [24] and so on. MIR217HG is the host gene of MIR217. Although the function of MIR217HG was undefined, there were multiple studies about function of MIR217 in different types of tumors. The studies of Su J, Wang H, Zhou W, Li H, Shen L, Guo J, Zhu Y, Wang X, Li J, N. A. Schultz et al respectively linked MIR217 to hepatocellular carcinoma [25], gastric Cancer [26], breast cancer [27], renal cell carcinoma [28], osteosarcoma [29], lung cancer [30], glioma [31], esophageal carcinoma [32], ovarian cancer [33], pancreatic cancer [8] et al.

In our research, the newly-diagnosed patients were selected as cases, which could effectively avoid the Neyman bias. Given that the prevalent patients are more likely to change the exposure status of environmental factors and living habits, compared with the newly-diagnosed patients, which is beneficial to survive with lung cancer, the prevalent patients shouldn’t be selected as the studying subjects. The cases and controls were selected from the same hospital, which could effectively avoid the Berkson bias. The observed genotype frequencies of rs1347093, rs1397529 among controls was in agreement with Hardy-Weinberg equilibrium, which means our studying subjects have good representativeness. In the present study, we carried out our studies, adjusting by gender, age and smoking. Above all of these could effectively improve the reliability of the results.

Our study firstly find that rs1347093, rs1397529 polymorphisms are associated with lung cancer risk in northeast Chinese population. However, some limitations should be considered before drawing a conclusion. Firstly, the present study is a hospital-based study, in which subjects in the control group are from the medical examination centers of the hospitals where our cases are selected. Therefore, controls may not be representative for the whole Chinese population very well. Secondly, in this study, all the subjects are from northeast China, thus a more diversified population should be selected to prove this results in the future study.

## MATERIALS AND METHODS

### Subject data collection

We carried out the hospital-based case-control study in Shenyang city which is located in the northeast China. Our study subjects consisted of 825 cases and 766 controls. All cases were diagnosed newly as lung cancer by the professional pathologists between March 2010 and January 2014. At the same time, we randomly selected people without a history of cancer as controls in the same hospital. In addition, we defined environmental exposure on the basis of our previous study [34]. Subjects who smoke more than 100 cigarettes in their lifetime were defined as smokers. Cases and controls were all unrelated ethic Han-Chinese people who lived in northeast and they had signed informed consent forms.

### DNA genotyping

Firstly, we obtained a 5ml venous blood samples from every participant and isolated genomic DNA by phenol-chloroform method. A 7500 Fast Real-time PCR system (Applied Biosystems, Foster City, CA, USA) performed SNP genotyping to design PCR Taqman primers and probes (assay ID C___2076188_10 for rs1347093 and C___289707_10 for rs1397529), using Applied Biosystems (CA, USA). The reaction conditions of the quantitative real-time PCR (q PCR) was as follows: 10min at 95°C following by 47 cycles of 92°C for 30s and 1min at 60°C. An ABI 7500 FAST Real-Time PCR System read the results of reaction, using the Sequence Detection Software. For the sake of quality control, the researchers were blinded to grouping state of subjects and we randomly selected 10% samples to carry out genotyping a second time. The results of the duplicated genotyping were 100% consistent with the former ones.

### Statistical analysis

T test and χ^2^ test were carried out to examine the difference of demographic variables and smoking status between cases and controls. Hardy-Weinberg equilibrium (HWE) was examined by a goodness-of-fit χ^2^ test in the control group. The odds ratios (OR) and 95% confident intervals (95% CI) were calculated by unconditional logistic regression analysis to estimate the relationship between SNP and the susceptibility of lung cancer. Multiplicative model and addictive model were conducted to evaluate the interaction between gene polymorphism and smoking status on lung cancer susceptibility. In multiplicative model, The odds ratios (OR) and 95% confident intervals (95%CI) were calculated in the logistic regression model to estimate the interaction. In addictive model, RERI (Relative Excess Risk due to Interaction), AP (Attributable Proportion due to Interaction), and S (Synergy Index) was used to estimate the interaction. When the 95%CI of S didn’t include 1 and the 95%CI of RERI and AP didn’t include 0, there was statistically significant interaction [35]. Almost analyses were adjusted by age, gender and smoking. All above analyses were two-sided, and were carried out by SPSS software (vision 20.0) unless specified. The criterion of statistical significance was defined as P<0.05.

## CONCLUSIONS

The results of the present study demonstrate that significant association between rs1347093, rs1397529 polymorphism and susceptibility to lung cancer may exist in northeast Chinese population. There is no significant interaction between rs1347093, rs1397529 polymorphism and smoking on susceptibility of lung cancer.

## References

[1] Siegel RL, Miller KD, Jemal A (2015). Cancer statistics, 2015. CA Cancer J Clin.

[2] Siegel RL, Miller KD, Jemal A (2017). Cancer statistics, 2017. CA Cancer J Clin.

[3] Torre LA, Bray F, Siegel RL, Ferlay J, Lortet-Tieulent J, Jemal A (2015). Global cancer statistics, 2012. CA Cancer J Clin.

[4] Jha P (2009). Avoidable global cancer deaths and total deaths from smoking. Nat Rev Cancer.

[5] Albright F, Teerlink C, Werner TL, Cannon-Albright LA (2012). Significant evidence for a heritable contribution to cancer predisposition: a review of cancer familiality by site. BMC Cancer.

[6] Brennan P, Hainaut P, Boffetta P (2011). Genetics of lung-cancer susceptibility. Lancet Oncol.

[7] Yin Z, Cui Z, Ren Y, Xia L, Li H, Zhou B (2017). MiR-146a polymorphism correlates with lung cancer risk in Chinese nonsmoking females. Oncotarget.

[8] Schultz NA, Werner J, Willenbrock H, Roslind A, Horn T, Wøjdemann M, Johansen JS (2011). MicroRNA expression profiles associated with pancreatic cancer. J Clin Oncol.

[9] Szafranska AE, Doleshal M, Edmunds HS, Gordon S, Luttges J, Munding JB, Barth RJ, Gutmann EJ, Suriawinata AA, Pipas JM, Tannapfel A, Korc M, Hahn SA (2008). Analysis of microRNAs in pancreatic fine-needle aspirates can classify benign and malignant tissues. Clin Chem.

[10] Nishida K, Hirano T (2003). The role of Gab family scaffolding adapter proteins in the signal transduction of cytokine and growth factor receptors. Cancer Sci.

[11] Gu H, Neel BG (2003). The ‘Gab’ in signal transduction. Trends Cell Biol.

[12] Burdon T, Stracey C, Chambers I, Nichols J, Smith A (1999). Suppression of SHP-2 and ERK signalling promotes self-renewal of mouse embryonic stem cells. Dev Biol.

[13] Holgado-Madruga M, Emlet DR, Moscatello DK, Godwin AK, Wong AJ (1996). A Grb2-associated docking protein in EGF- and insulin-receptor signalling. Nature.

[14] Nishida K, Yoshida Y, Itoh M, Fukada T, Ohtani T, Shirogane T, Atsumi T, Takahashi-Tezuka M, Ishihara K, Hibi M, Hirano T (1999). Gab-family adapter proteins act downstream of cytokine and growth factor receptors and T- and B-cell antigen receptors. Blood.

[15] Yamasaki S, Nishida K, Yoshida Y, Itoh M, Hibi M, Hirano T (2003). Gab1 is required for EGF receptor signaling and the transformation by activated ErbB2. Oncogene.

[16] Fan Y, Yang F, Cao X, Chen C, Zhang X, Zhang X, Lin W, Wang X, Liang C (2016). Gab1 regulates SDF-1-induced progression via inhibition of apoptosis pathway induced by PI3K/AKT/Bcl-2/BAX pathway in human chondrosarcoma. Tumour Biol.

[17] Bai R, Weng C, Dong H, Li S, Chen G, Xu Z (2015). MicroRNA-409-3p suppresses colorectal cancer invasion and metastasis partly by targeting GAB1 expression. Int J Cancer.

[18] Chen D, Zhong F, Chen Y (2017). Association of calcium/calmodulin-dependent protein kinase kinase1 rs7214723 polymorphism with lung cancer risk in a Chinese population. Biosci Rep.

[19] Xie K, Chen M, Zhu M, Wang C, Qin N, Liang C, Song C, Dai J, Jin G, Shen H, Lin D, Ma H, Hu Z (2017). A polymorphism in miR-1262 regulatory region confers the risk of lung cancer in Chinese population. Int J Cancer.

[20] Yin Z, Li H, Cui Z, Ren Y, Li X, Wu W, Guan P, Qian B, Rothman N, Lan Q, Zhou B (2016). Polymorphisms in pre-miRNA genes and cooking oil fume exposure as well as their interaction on the risk of lung cancer in a Chinese nonsmoking female population. Onco Targets Ther.

[21] Singh A, Singh N, Behera D, Sharma S (2017). Polymorphism in XRCC1 gene modulates survival and clinical outcomes of advanced North Indian lung cancer patients treated with platinum-based doublet chemotherapy. Med Oncol.

[22] Ortiz-Padilla C, Gallego-Ortega D, Browne BC, Hochgrafe F, Caldon CE, Lyons RJ, Croucher DR, Rickwood D, Ormandy CJ, Brummer T, Daly RJ (2013). Functional characterization of cancer-associated Gab1 mutations. Oncogene.

[23] Hu L, Liu R (2016). Expression of Gab1 is associated with poor prognosis of patients with epithelial ovarian cancer. Tohoku J Exp Med.

[24] Chang CH, Chan PC, Li JR, Chen CJ, Shieh JJ, Fu YC, Chen HC, Wu MJ (2015). Gab1 is essential for membrane translocation, activity and integrity of mTORCs after EGF stimulation in urothelial cell carcinoma. Oncotarget.

[25] Su J, Wang Q, Liu Y, Zhong M (2014). miR-217 inhibits invasion of hepatocellular carcinoma cells through direct suppression of E2F3. Mol Cell Biochem.

[26] Wang H, Dong X, Gu X, Qin R, Jia H, Gao J (2015). The microRNA-217 functions as a potential tumor suppressor in gastric cancer by targeting GPC5. PLoS One.

[27] Zhou W, Song F, Wu Q, Liu R, Wang L, Liu C, Peng Y, Mao S, Feng J, Chen C (2017). miR-217 inhibits triple-negative breast cancer cell growth, migration, and invasion through targeting KLF5. PLoS One.

[28] Li H, Zhao J, Zhang JW, Huang QY, Huang JZ, Chi LS, Tang HJ, Liu GQ, Zhu DJ, Ma WM (2013). MicroRNA-217, down-regulated in clear cell renal cell carcinoma and associated with lower survival, suppresses cell proliferation and migration. Neoplasma.

[29] Shen L, Wang P, Yang J, Li X (2014). MicroRNA-217 regulates WASF3 expression and suppresses tumor growth and metastasis in osteosarcoma. PLoS One.

[30] Guo J, Feng Z, Huang ZA, Wang H, Lu W (2014). MicroRNA-217 functions as a tumour suppressor gene and correlates with cell resistance to cisplatin in lung cancer. Mol Cells.

[31] Zhu Y, Zhao H, Feng L, Xu S (2016). MicroRNA-217 inhibits cell proliferation and invasion by targeting Runx2 in human glioma. Am J Transl Res.

[32] Wang X, Li M, Wang Z, Han S, Tang X, Ge Y, Zhou L, Zhou C, Yuan Q, Yang M (2015). Silencing of long noncoding RNA MALAT1 by miR-101 and miR-217 inhibits proliferation, migration, and invasion of esophageal squamous cell carcinoma cells. J Biol Chem.

[33] Li J, Li D, Zhang W (2016). Tumor suppressor role of miR-217 in human epithelial ovarian cancer by targeting IGF1R. Oncol Rep.

[34] Ren Y, Yin Z, Li K, Wan Y, Li X, Wu W, Guan P, Zhou B (2015). TGFβ-1 and TGFBR2 polymorphisms, cooking oil fume exposure and risk of lung adenocarcinoma in Chinese nonsmoking females: a case control study. BMC Med Genet.

[35] Quan X, Yin Z, Fang X, Zhou B (2017). Single nucleotide polymorphism rs3124599 in Notch1 is associated with the risk of lung cancer in northeast Chinese non-smoking females. Oncotarget.

